# Editorial: Rising stars in reproduction: 2021

**DOI:** 10.3389/fendo.2023.1151313

**Published:** 2023-02-22

**Authors:** Giuliano Bedoschi

**Affiliations:** University of Sao Paulo, Ribeirao Preto Medical School, Department of Gynecology and Obstetrics, Reproductive Medicine Division, Ribeirao Preto, Brazil

**Keywords:** fertility preservation, *in vitro* fertilization, embryonic development, ovulation induction, spermatozoa, infertility, oncofertility, endometriosis

The field of reproductive medicine is rapidly evolving, with the potential to drastically impact the lives of individuals and families worldwide. However, the constant emergence of new technologies and treatments can make it challenging to stay abreast of the latest advancements. Additionally, it is important to acknowledge that access to reproductive healthcare and technology remains disparate across different nations (1). Despite these challenges, there are a number of rising researchers in the field who are making significant contributions and warrant attention. In this editorial, we will examine the work of several of these pioneering investigators in the area of reproductive medicine.

The study published by Mateo-Otero et al. investigated the presence of the protein Aldose reductase B1 (AKR1B1) in sperm and its relationship to sperm quality, functionality, and fertilizing ability. The research discovered the presence of AKR1B1 in both epididymal and ejaculated sperm, with comparable levels, and established a negative correlation between the levels of AKR1B1 in sperm and kinematic parameters and intracellular calcium, and a positive correlation with the proportion of sperm possessing distal cytoplasmic droplets after preservation. Additionally, lower levels of AKR1B1 in sperm were associated with better reproductive outcomes such as higher fertilization rates and better embryo development. The research indicates that AKR1B1 is present in both epididymal and ejaculated sperm and may play a role in the maturation process. Additionally, the levels of AKR1B1 seem to impact sperm’s ability to withstand preservation and fertilization, with higher levels potentially being related to greater resilience in these areas.

Zhu et al. used bibliometric analysis to assess the worldwide scientific production in the field of infertility and psychology over the past two decades. The study identified a total of 151 articles, and found an increasing trend of publications from 2001 to 2021, with a relatively stable trend in the past eight years. The leading journal was Human Reproduction, and the most prolific author was Boivin J, who had the highest h-index. The United Kingdom and Cardiff University produced the highest number of scientific works in this field. The study also found active cooperation between countries and institutions and identified the key topics of interest, including women, *in-vitro* fertilization, infertility, couples, and the impact of infertility. The study provides an overview of the current research status in this field and highlights that infertility and psychology are widely recognized as important research areas and are likely to continue to attract attention from researchers.

The study published by Roos et al. aimed to investigate the relationship between ovarian sensitivity index (OSI) and *in vitro* fertilization (IVF) outcomes. The OSI was employed to gauge the ovary’s response to gonadotropin stimulation prior to IVF. The study found that a reduction in responsiveness to gonadotropins, also known as hypo response, may lead to a low number of retrieved oocytes and failure of IVF. The study suggests that the underlying mechanisms of hypo response, especially in young women and those with advanced maternal age, are not fully understood. The research utilized single-cell analysis techniques to examine the variability of ovarian follicular cell types and their gene expression patterns among patients with hyporesponsiveness to gonadotropin stimulation during IVF. The goal was to improve their chances of live birth through IVF. The study found 12 molecular pathways that were differently regulated in hypo- and normoresponder groups, and that the prevalence of three cell clusters was significantly dissimilar between these groups, signifying their role in hyporesponsiveness. The findings offer novel perspectives on the differences in gene expression of follicular cells between hypo- and normoresponders and contribute to the understanding of variability of cell types in human preovulatory follicles as revealed by single-cell analysis of follicular fluid cells.

Morcillo i Soler et al. investigates the effect of physical and biochemical factors on the formation of sperm bundles, which is a mechanism of sperm competition observed in several species. The research explored various factors that may impact the occurrence of sperm bundling, such as the viscosity of the fluid, the process of swim-up, post-thaw incubation duration, and media components that enhance capacitation. The findings indicate that while fluid viscosity does not appear to have a significant effect on the severity of sperm bundling, an increase in sperm bundling was observed when utilizing swim-up, longer post-thaw migration times, and suppressed capacitation. The study concludes that sperm bundling is the result of hydrodynamic and adhesive interactions between cells, which tend to occur more frequently during extended incubation periods.

The study published by Kusama et al. aimed to investigate the role of a protein called SERPINA1 in the development of endometriosis, a condition marked by the existence of endometrial tissue that is swollen and contains excessive fibrous tissue, which is found outside the uterus. The study used a mouse model of endometriosis and found that decreased SERPINA1 expression in endometriosis-like lesions exacerbates inflammation in these lesions. The study analyzed the effect of SERPINA1 reduction on endometrial stromal cells (ESCs) using RNA sequencing techniques and discovered an increase in the expression of several toll-like receptor (TLR) related factors. The results revealed that the silencing of SERPINA1 led to heightened expression of TLR3 and TLR4, as well as key components of the TLR signaling pathway, including MYD88, IRAK1/4, interleukin (IL)-1β, and interferon (IFN)-β. The research indicates that a decrease in SERPINA1 expression stimulates the production of inflammatory factors by ESCs, which are then linked to TLR3/4, IL-1β, and IFN-β signaling. The conclusion of the study is that controlling the levels of SERPINA1 within ESCs could be a potential strategy for reducing inflammation in endometriotic lesions.

Cannarella et al. examines the presence and potential role of the protein Insulin-like growth factor 2 (IGF2) in human sperm. The study discovered the existence of IGF2 protein in sperm specimens and observed that it is located in the equatorial and post-acrosomal segment with varying levels of expression. The study also looked at the effect of IGF2 on the proliferation and gene expression of mitogens and hormone receptors in Sertoli cells (SCs). The results suggest that IGF2 suppresses the proliferation and gene expression of mitogens and hormone receptors in SCs, suggesting that IGF2 could be involved in the communication process between sperm and Sertoli cells during the formation of sperm.

In conclusion, this editorial article has highlighted a selection of recent research studies that provide new insights into a range of reproductive medicine topics. From investigating the presence and role of proteins in sperm quality and fertilizing ability to exploring the relationship between ovarian sensitivity and *in vitro* fertilization outcomes, these studies offer a glimpse into the cutting-edge research being conducted in the field of reproductive medicine. Additionally, the studies on sperm bundle formation and the role of specific proteins in endometriosis further expand our understanding of the underlying mechanisms at play in these conditions. As research in these areas continues to evolve, we can expect to see an increasing number of innovative treatments and diagnostic tools to improve the outcomes for couples struggling with infertility.

## Author contributions

GB contributed to the conceptualization, drafting, and revision of the manuscript. GB has approved the final version of the manuscript for submission.
